# Growth faltering in early infancy: highlights from a two-day scientific consultation

**DOI:** 10.1186/s12919-020-00195-z

**Published:** 2020-09-15

**Authors:** Satinder Aneja, Praveen Kumar, Tarun Shankar Choudhary, Akanksha Srivastava, Ranadip Chowdhury, Sunita Taneja, Nita Bhandari, Abner Daniel, Purnima Menon, Harish Chellani, Rajiv Bahl, Maharaj Kishan Bhan

**Affiliations:** 1 School of Medical Sciences and Research, Sharda University, Greater Noida, India; 2 Department of Pediatrics, Lady Hardinge Medical College and Associated Kalawati Saran Children’s Hospital, New Delhi, India; 3 Knowledge Integration and Translational Platform (KnIT) at the Centre for Health Research and Development, Society for Applied Studies, New Delhi, India; 4 Nutrition Section, UNICEF India Country Office, New Delhi, India; 5 Poverty, Health, and Nutrition Division, International Food Policy Research Institute, New Delhi, India; 6 Department of Pediatrics, Vardhman Mahavir Medical College and Safdarjung Hospital, New Delhi, India; 7 Department of Maternal, Newborn, Child and Adolescent Health, World Health Organisation, Geneva, Switzerland; 8 National Science Professor, Indian Institute of Technology, New Delhi, India; 9 Knowledge Integration and Translational Platform (KnIT), Biotechnology Industry Research Assistance Council (BIRAC), New Delhi, India

**Keywords:** Growth faltering, Severe wasting, Severe acute malnutrition, Early infancy

## Abstract

Faltering of growth in early life has been recognized as a public health challenge among Indian babies. A two-day consultation on growth faltering in early infancy was organized to examine the data and evidence on identification and management of early growth failure and to identify knowledge gaps and future areas of research. The consultation was supported by the Biotechnology Industry Research Assistance Council (BIRAC), the Indian Academy of Pediatrics (Nutrition Chapter), Vardhman Mahavir Medical College and Safdarjung Hospital, and the Society for Applied Studies. It brought together researchers, clinicians, policy makers and program managers.

## Key highlights

1. Available data from national surveys and research studies highlights that early growth faltering remains a significant concern in India.
2. Evidence is inconclusive on the most robust anthropometric parameters for identification of growth failure in young infants. Studies seem to suggest that weight-for-age Z (WAZ) may be the best predictor of mortality among infants less than six months of age. Use of WAZ could operationally simplify the community level assessment of at-risk infants in this age group.
3. Current national guidelines for management of growth faltering among infants less than six months of age are based on low or very low-quality evidence.
4. More research is needed on facility as well as community-based management of growth faltering in this age group. Identifying the window of opportunity for maximizing catchup growth in pre-term and small for gestational age babies is a priority.
5. Research is needed to assess how the health system needs to be re-organized for facilitating optimum care of small and sick babies through the continuum of facility births and follow up care at home. Comprehensive models for care of small and sick newborns extending beyond neonatal period are needed. Models incorporating interventions like no separation policy of mothers and newborns, Kangaroo Mother Care, with co-delivery of postnatal care and Early Newborn Care should be tested for scale up in program settings.

Overall, the aim of the research agenda ahead is to identify scalable effective actions to prevent and recuperate from growth faltering that also help in improving survival, health and neurodevelopment, without contributing to adverse long-term metabolic risks.

## Background

Growth faltering among infants less than six months of age has not received adequate attention from a prevention, timely detection and management perspective [1]. A large proportion of early growth faltering can be attributed to low birth weight which is a result of preterm birth and/or intrauterine growth restriction. Around 6 million babies in India are born low birth weight and the prevalence of stunting, wasting and underweight in children under six months of age is 20.1, 31.9 and 26.7% respectively [2]. Further, a substantial number of children under six months of age have extreme deviations in growth, as 9.5% have severe stunting, 14.9% have severe wasting and 9.5% have severe underweight [2].

Improved understanding of growth processes in the first six months of life, is critical to prevention, early diagnosis and appropriate management of growth faltering. Evidence based guidelines are necessary on the nutritional needs of preterm, small for gestational age and low birth weight infants who are at higher risk of growth faltering in the first 6 months of life. Attainment of full potential for physical growth and development, without being exposed to the risk of adiposity and cardiovascular diseases later in life is desirable [3–5].

A two-day meeting of subject experts, to discuss the current understanding of growth patterns and their deviation in early infancy, was organized in October 2018, at New Delhi, India. The consultation was organized by the Biotechnology Industry Research Assistance Council (BIRAC), the Indian Academy of Pediatrics (Nutrition Chapter), Vardhman Mahavir Medical College and Safdarjung Hospital, and the Society for Applied Studies. This two-day consultation witnessed engaging discussions among subject experts on the identification and appropriate management of infants who have or are vulnerable to growth failure during the first six months of life. A range of discussions were also held on the research agenda related to identification, prevention and management. In this article the authors present the discussions and learnings from the consultation around the following four themes:

- Growth failure and its epidemiology in early infancy
- Guidelines and current practices on diagnosis and management of growth failure in early infancy
- Nutritional requirements for catch-up growth in young infants
- Promoting survival, growth and development of low birth weight babies and other high-risk groups

### Growth failure and its epidemiology in early infancy

Infants less than six months old are an important group as they carry the highest risk of mortality and morbidity [1]. In addition, studies show that early growth faltering is associated with an increased risk of chronic diseases in later life [6].

Reliable estimates at national and state level are necessary for planning programs for prevention, identification and management of growth faltering. The estimates for the burden of stunting, wasting and underweight from nationally representative surveys like National Family Health Survey (NFHS) [2] and Comprehensive National Nutrition Survey (CNNS) [7] are however, higher than those reported from cohorts and randomized trials.

The reliability of anthropometric measurements in large surveys is also an area of concern as the measurement of recumbent length and weight, especially among young infants is challenging and error prone [8]. These can have substantial effects on cut-off-based criteria using Z scores i.e. < −2SD or < −3SD for identifying growth faltering. The anthropometric indicator for identifying high risk infants in the first 6 months of life should be guided by outcomes like mortality and morbidity in the first year of life and, risk of undernutrition at 6 and 12 months of life. The use of the terminology ‘severe wasting’ may be more appropriate than severe acute malnutrition (SAM) as the condition is not acute and in many cases several newborns are “SAM” at birth.

Early growth faltering may result from a mix of factors like low birth weight (preterm birth with or without small for gestational age), suboptimal feeding practices, poor maternal nutrition, maternal illness, lack of responsive care giving, poor sanitation and environmental enteropathy. Low birth weight, non-utilization of nutrition support from the ICDS program [9] and anthropometric measurements in the summer and monsoon seasons have been found to be associated with an increased risk of growth faltering [10].

High quality evidence on the best anthropometric indicators for predicting the risk of mortality among infants less than six months with growth faltering is lacking. Secondary data analysis of a multi-country (India, Ghana and Peru) randomized controlled trial shows that weight-for-age Z score (WAZ) has highest sensitivity and specificity for predicting mortality among infants less than six months of age [11]. Validating these findings in other settings is necessary for guideline formulation. These would also be of relevance to policymakers as measurement of weight is less error prone in field settings, compared to assessment of length/height [12, 13]. Body mass index (BMI) for age Z can be explored as an alternative indicator in the first six months of life, but this will also be susceptible to error in length measurement. More evidence would be needed before these are used in programs.

Clinical decision making and monitoring growth in the first six months of life may also be based on growth velocity i.e. length gain and weight gain and shifts in LAZ, WAZ, WLZ in addition to the WHO 2006 growth standards. Growth velocity charts are important to evaluate the growth of a subject and are available for height and weight, in both boys and girls. Height or weight velocity is a variable derived from the measurement of height or weight at different times and represents the increase in height or weight during a specified time period. Faltering in height gain and/or weight gain beyond a certain cut-off usually taken as 3rd centile, should warrant appropriate measures like additional nutritional support and detailed clinical, biochemical and endocrinological evaluation [14].

### Guidelines and current practices on diagnosis and management of growth failure in early infancy

The current guidelines for the identification and management of growth faltering among infants under 6 months of age are primarily focused on severe acute malnutrition. This is because severe acute malnutrition in infants under 6 months is associated with high risk of mortality [15]. The WHO has published specific recommendations for identification and management of severe malnutrition among infants less than six months of age [16]. The cut-off for identification being used currently is a WLZ score < −3SD of the WHO 2006 growth reference or any pitting edema. Recent weight loss or failure to gain weight; any medical or social issue needing more detailed assessment or intensive support are also to be used for assessment of the need for inpatient care.

Infants who are less than 6 months of age with severe acute malnutrition should receive the same general medical care as infants with severe acute malnutrition who are 6 months of age or older. Feeding approaches for infants who are less than 6 months of age with severe acute malnutrition prioritize establishing, or re-establishing, effective exclusive breastfeeding by the mother or other caregiver. Supplementary suckling approaches, where feasible, are prioritized. For infants with severe acute malnutrition but no edema, expressed breast milk should be given, and, where this is not possible, commercial (generic) infant formula or F-75 or diluted F-100 may be given, either alone or as the supplementary feed together with breast milk. For infants with severe acute malnutrition and edema, infant formula or F-75 should be given as a supplement to breast milk.

Transfer to outpatient care is recommended when all clinical conditions or medical complications, including edema, are resolved, and the infant has good appetite, is clinically well and alert, and weight gain on either exclusive breastfeeding or replacement feeding is satisfactory, e.g. above the median of the WHO growth velocity standards or more than 5 g/kg/day for at least 3 successive days, and the infant has been checked for immunizations and other routine interventions, and the mother or caregiver is linked with needed community-based follow-up and support. Infants who are less than 6 months of age can be discharged from all care when they are breastfeeding effectively or feeding well with replacement feeds, and have adequate weight gain, and have a WLZ ≥ −2SD.

For infants who are less than 6 months of age with severe acute malnutrition and who do not require inpatient care, or whose caregivers decline admission for assessment and treatment, the following is done:

- counselling and support for optimal infant and young child feeding should be provided, based on general recommendations for feeding infants and young children, including for low birthweight infants.
- weight gain of the infant should be monitored weekly to observe changes; if the infant does not gain weight or loses weight while the mother or caregiver is receiving support for breastfeeding, then he or she should be referred to inpatient care.
- assessment of the physical and mental health status of mothers or caregivers should be promoted, and relevant treatment or support provided.

The guidelines on admission criteria, inpatient care guidelines and shifting to outpatient care, are all based on low or very low-quality evidence. More evidence is needed on the identification and management (facility and community) of severely wasted infants less than six months.

The Government of India has also published guidelines for the management of SAM which includes facility-based care of infants under 6 months of age. Supporting supplementary suckling is the cornerstone of recommended management approaches in this age group and F-75 therapeutic food is recommended only if re-lactation is not possible [17].

Current facility-based management of SAM for infants relies on providing local preparations of starter and catch-up diets, along with provision of micronutrient supplements. This is operationally challenging to deliver. Thus, the Ministry of Health, Government of India needs to consider adoption of globally recommended F-75 and diluted F-100 standard preparations to be used for facility-based care of infants with severe acute malnutrition for short term use [16]. The feasibility of establishing this protocol and adapting for Indian public health facilities, as necessary should be prioritized. This is especially important because the burden of wasting and severe wasting in India is high at birth and among infants less than six months of age. With increasing focus on the critical age group of less than six months, a larger proportion of admissions to NRCs would be for infants in this age group.

### Nutritional requirements for catch-up growth in young infants

The role of optimal breastfeeding practices, in preventing growth failure among infants less than six months of age, is central. Providing adequate breastfeeding support for mothers through counselling services by trained and skilled personnel in facilities and at the community level is critical. In infants less than six months of age with SAM, establishment or re-establishment of exclusive or partial breastfeeding should be attempted. In NRCs, breastfeeding problems can be successfully managed through lactational counselling, supportive care and supplementary suckling techniques. Supporting maternal psychosocial health is important for better nutrition outcomes in the first six months of life [18, 19].

Identifying the best alternatives to breastfeeding in cases of maternal milk insufficiency despite intensive support by lactational counsellors is very important. Supplementary suckling technique (SST), provision of donor human milk, formula feeds, diluted F-100 and F-75, are the options that can be considered for use in this age group. The current strength of 30 human milk banks in India is grossly inadequate given the high burden of preterm and small for gestational age babies [20]. The choice of F-75 and diluted F-100 as standard preparation for in-patient care of SAM in infants less than six months of age needs to be assessed, along with the criteria for deciding recovery and discharge. A feasible model for lactational support in program settings in terms of personnel, training and placement of counsellors, based on best practices globally should be identified. Recommended Nutritional intake/Recommended Dietary Allowance (RDA) guidelines for preterm are well defined, but these have not been fully evaluated for small for gestational age infants [21]. Evidence on the need for micronutrient supplementation and fortification of breastmilk or infant formula for all infants with severe wasting, or for a subset is required before changes to the guidelines for lactation support are made.

There are concerns about rapid catchup growth and risk of adiposity and cardiovascular diseases in later life, yet the evidence to date is limited and inconclusive [3–5]. This hypothesis has important implications for guidelines and policies that aim to strike a balance between short term and long-term outcomes. Therefore, more evidence base of high quality is further warranted in this domain. Nonetheless, catchup growth has been shown to reduce mortality and morbidity outcomes in short term and neurodevelopment in later life. Identifying the window of opportunity for maximizing appropriate catchup is a priority. Available evidence points towards the need to prioritize the first 2–4 months of age as the period for maximum potential for catch-up.

### Promoting survival, growth and development of newborns at risk of growth faltering

Given the high burden of growth faltering in India, the health system should focus on prevention, with appropriate mechanisms to take care of infants with growth faltering. Infants born preterm or small for gestational age or low birth weight are at high risk of growth faltering. The survival, growth and development of these infants requires a continuum of care approach wherein there is appropriate linkage between immediate post-natal care in facilities and subsequent follow up in community settings.

Several models of keeping non critically ill small babies along with their mothers in a single unit or ward at a facility are currently being evaluated in India. Successful models of no mother-child separation of small babies should be scaled up. According to national guidelines, district level hospitals should establish a kangaroo mother care unit for low birth weight infants who do not require care in sick newborn care units (SNCU). The scale up of this has been limited though [22, 23]. Integrating good infrastructure, treating mothers with respect, lactation support, kangaroo mother care (KMC) and counselling of mothers at the time of discharge are important. Care for sick newborns should use a family centered approach with active participation of the infant’s family [24]. National programs should aim to achieve reduction in mortality, improving follow up rates of these newborns in community, achieving sustained exclusive breastfeeding and continuing KMC at home after discharge.

There is ample evidence suggesting that large numbers of children with SAM who do not have medical complications (85–90% of all SAM children) can be treated in their communities without being admitted to a health facility [17]. Besides, children managed at specialized units located at health facilities also need to be followed up at their households and communities after being discharged for continued care and support; and to prevent the relapse.

Therefore **community-based efforts**, which complement and link to facility basedinterventions should be put in place simultaneously. It must be recognized that although treatment is urgently needed for those who are severely undernourished, preventing child undernutrition is critical. NRCs will reduce child mortality but will not improve the general nutritional status of children in the community. From the perspective of health sector, the most important intervention is promotion of appropriate infant and young child feeding and nutrition practices and related maternal undernutrition. Given that there are many social determinants of poor child growth, including early childbearing, poor sanitation, poverty, and parental education, efforts to prevent child undernutrition must also improve these conditions [25–27].

Programs and research should explore the feasibility of community-based management of uncomplicated SAM among infants less than six months of age. Some challenges that were discussed at the consultation were that linkage between inpatient and outpatient/community care for severe acute malnutrition are critical. The package of care should be simple and easy to implement and should link seamlessly with existing government programs and interventions such as home based newborn care, home based young infant care, kangaroo mother care, growth monitoring and the Integrated Management of Neonatal and Childhood Illness [28–31]. The goal is to ensure reduced mortality and morbidity along with improved development.

## Summary

The consultation held in India highlighted a range of issues related to identification and management of young infants, under six months of age. Below, we summarize the major areas of consensus as well as areas that require more action, and more research.

### Indicators for identification of growth faltering among infants under six months of age

There is a lack of consensus on the best indicator for predicting risk of mortality among infants less than six months of age with growth faltering. Limited evidence suggests that WAZ, can be used for identification and follow-up of at-risk infants. The consultation group did not arrive at a conclusion about the best choice but noted that the issue of indicator performance for identification of risk of mortality should be explored further, using existing datasets or through new studies, if necessary. A persistent deterioration in growth velocity i.e. length gain and weight gain and shifts in LAZ, WAZ, WLZ score can be useful for clinical decision-making, complementing the cut-off-based approach. Good clinical measurement of growth and developmental outcomes at multiple points in time is necessary. Overall, the group agreed that simple, easy to measure indicators were important to help with early identification and management of growth faltering and its monitoring in community settings.

In addition, generating reliable data on the burden of growth faltering in early life using periodic population level surveys is necessary for guiding geographic or sub-group targeting of public policy actions.

### Management of growth faltering among infants under six months of age

A range of policy guidelines and existing government programs provide a platform to strengthen the care of newborns who are vulnerable to early growth faltering. However, these guidelines for clinical management of growth faltering among infants less than six months of age are based on low or very low-quality evidence.

Evidence is needed regarding the most effective approaches for strengthening effective lactation support for young infants and for assessing, where needed, effective and safe alternatives for breastfeeding. In addition, nutrient requirements for optimal physical growth of SGA and LBW infants, and guidelines for micronutrient supplementation or fortification should be established as SGA and LBW infants are different from preterm LBW infants in several ways [31].

In addition to the clinical management issues noted above, given the high burden of severe wasting in early infancy, the feasibility, impact and cost-effectiveness of community-based management for uncomplicated cases among infants less than six months of age should be explored in trials. Successful models should then be tested for the potential to scale up into existing delivery systems.

### Prevention of growth faltering at birth and among infants less than six months

Preventing undernutrition among infants under 6 months of age needs to be viewed in context of mother-infant dyad as fetal growth and growth during period of breast feeding is intricately related to mother’s physical and mental health, and other social and economic conditions of the households and communities which are at risk of having wasted infants. The birth weight of infant is an important predictor of growth and survival which in turn is dependent upon health and nutrition of women before and during pregnancy. There is need for focusing on interventions which improve maternal health and nutrition and integrate these with interventions for child health. Ensuring adequate preventive and promotive care for growth and development of infants who are vulnerable at birth is a priority; this requires implementing, with quality all the existing programs in India’s policy framework. The health system actions need to be re-organized to facilitate optimum care of small and sick babies through the continuum of facility birth and follow up care at home post-discharge. For instance, in the health system, the recent Home-Based Care for Young Child (HBYC) guidelines from the Government of India mandate 5 additional visits at 3, 6, 9, 12 and 15 months of age by the Accredited Social Health Activist (ASHA) for all children, in addition to the visits during the first six weeks of life [29, 32]; these visits should be used to test integration of actions related to prevention, identification and management of severe wasting. Available interventions for delivery by ASHA and Auxiliary Nurse Midwife (ANM) are targeted at the known risk factors for growth failure in early life. These include infant and young child feeding (IYCF) counselling, support for timely initiation of breastfeeding, improved lactational support, KMC in facilities and at home for low birth weight children. In addition to these, intensive lactation counselling support directed towards at-risk cases can be developed based on the best practices globally and experiences in India. Finally, in light of the social determinants, it is important to strengthen integrated action and convergence of interventions and programs across the domains of health, nutrition, WASH and early childhood development on vulnerable households and communities.

## Data Availability

Not Applicable.

## Abbreviations

ANM: Auxiliary Nurse Midwife
ASHA: Accredited Social Health Activist
BMI: Body Mass Index
cMAMI: Community Management of At-Risk Mothers and Infants
HBYC: Home-Based Care for Young Child
IMCI: Integrated Management of Childhood illness
IMNCI: Integrated Management of Neonatal and Childhood Illness
IYCF: Infant and Young Child Feeding
KMC: Kangaroo Mother Care
LBW: Low Birth Weight
MUAC: Mid Upper Arm Circumference
NFHS: National Family Health Survey
CNNS: Comprehensive National Nutrition Survey
NRC: Nutrition Rehabilitation Centers
RDA: Recommended Dietary Allowance
SAM: Severe Acute Malnutrition
SGA: Small for Gestational Age
SNCU: Sick Newborn Neonatal Care Unit
SST: Supplementary Suckling Technique
WASH: Water Sanitation and Hygiene
WAZ: Weight-for-age Z
WHO: World Health Organization
WHZ: Weight-for-height Z
WLZ: Weight-for-length Z
